# Twenty-five years of progress in Parkinson’s disease research: from dopamine replacement to biological definition and regenerative therapy

**DOI:** 10.1007/s00702-026-03308-8

**Published:** 2026-10-24

**Authors:** Juergen Winkler

**Affiliations:** Department of Molecular Neurology, University Hospital Erlangen, 91054 Erlangen, Germany

**Keywords:** Parkinson’s disease, α-synuclein, Biomarkers, Prodromal disease, Lysosome, Immunotherapy, Stem cells

## Abstract

The first quarter of the twenty-first century has transformed Parkinson’s disease (PD) from a predominantly motor, dopamine-deficiency syndrome into a heterogeneous multisystem disorder defined across clinical, genetic and molecular dimensions. This overview follows the trajectory of these advances from the historical foundations established prior 2000 to present concepts of prodromal disease, progression, biological classification and precision medicine. Epidemiological work has established PD as the fastest-growing major neurological disorder, while systematic characterization of non-motor symptoms has shifted diagnosis and attention beyond the nigrostriatal system in PD research. Imaging has progressed from exclusionary structural magnetic resonance imaging (MRI) to quantitative susceptibility, diffusion and molecular approaches, although no single imaging biomarker yet provides a stand-alone diagnosis of PD. The discovery of α-synuclein gene mutations and its presence as protein in Lewy bodies and neurites connected genetics, protein aggregation and selective neuronal vulnerability. Seed amplification assays (SAA) now enable robust detection of pathological α-synuclein in cerebrospinal fluid and have catalysed biologically anchored staging proposals. At the same time, negative phase 2 antibody against α-synuclein trials illustrate the challenges of target engagement, disease heterogeneity and intervention timing. Genetics and cell biology have placed lysosomal proteostasis, in particular glucocerebrosidase 1 (GBA1) dependent pathways, at the center of disease-modification strategies. Dopaminergic therapy remains extraordinarily effective symptomatically, with continuous delivery systems extending its utility for intermediate and late stages of the disease, whereas cell replacement has re-entered clinical development through standardized pluripotent-stem-cell-derived midbrain dopaminergic progenitors. The next phase of PD research will depend less on a single unifying mechanism than on integrating molecular state, the pattern of neuronal injury, clinical phenotype and individual trajectory to select the right intervention at the right biological stage.

## Introduction: the foundations established before 2000

**Fig. 1:**
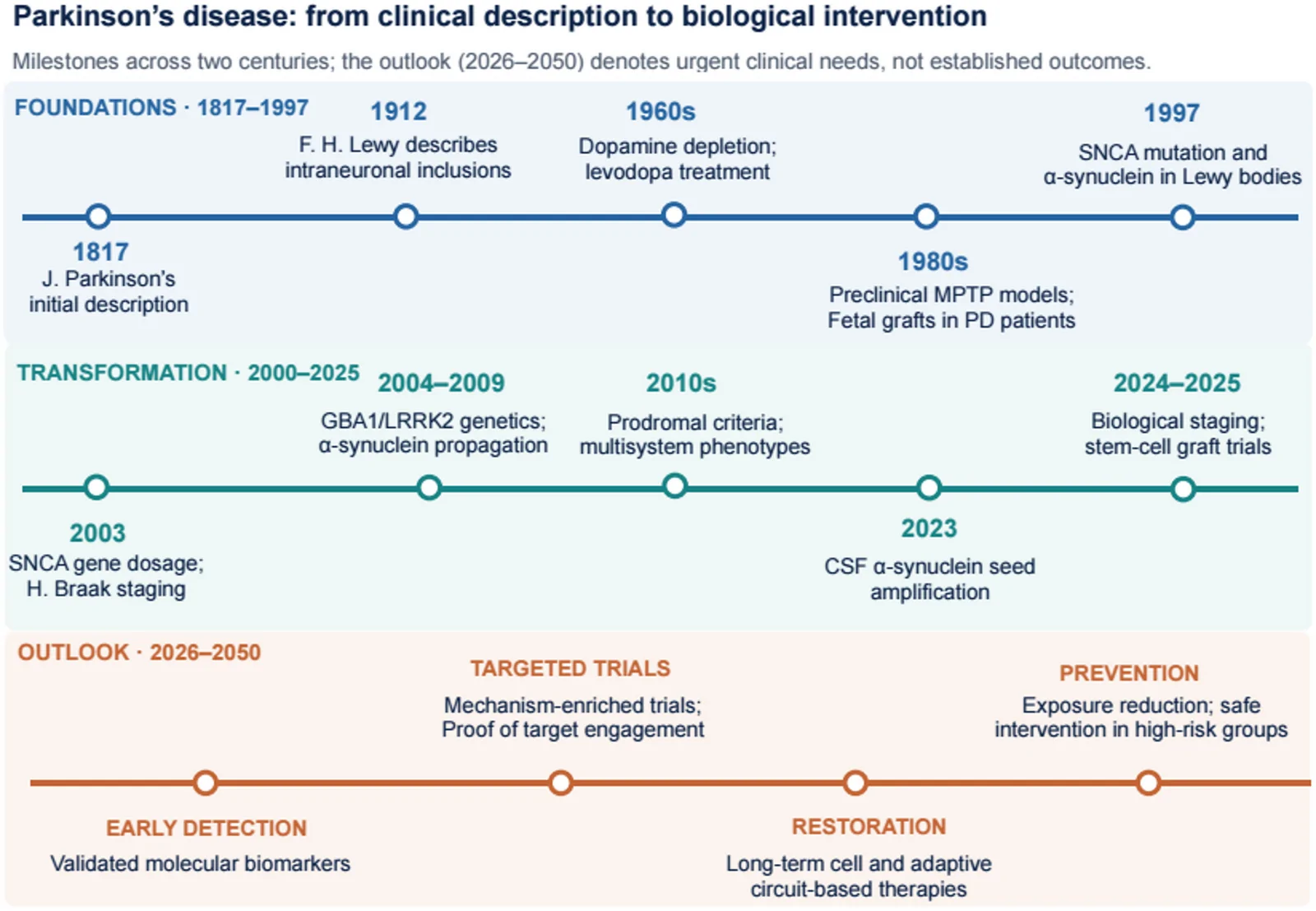
Parkinson’s disease: from clinical description to biological intervention. Milestones across two centuries: FOUNDATIONS (1817–1997), TRANSFORMATION (2000–2025), and OUTLOOK (2026–2050). The latter one denotes urgent clinical needs, not milestone or established outcomes. PD, Parkinson’s disease; MPTP, 1-Methyl-4-phenyl-1,2,3,6-tetrahydropyridine; SNCA, gene of α-synuclein; GBA1, glucocerebrosidase 1; LRRK2, leucin-rich repeat kinase 2; CSF, cerebrospinal fluid

Modern PD research rests on several key discoveries made long before the period reviewed here. James Parkinson’s 1817 precise clinical description, the identification of substantia nigra degeneration and Lewy bodies by Friedrich H. Lewy, and the demonstration in the late 1950s and early 1960s that striatal dopamine is profoundly depleted established the longstanding neuroanatomical and biochemical framework of the disease (Fig. 1). The ensuing introduction of levodopa remains one of neurology’s most successful examples of mechanism-based symptomatic treatment. By 2000, however, the field was already moving beyond dopamine deficiency. The identification of the A53T SNCA mutation in familial PD pedigrees (Polymeropoulos et al. 1997) and the demonstration that α-synuclein is a major constituent of Lewy bodies (Spillantini et al. 1997) linked a rare genetic form to the neuropathology of the most frequent phenotype, sporadic PD. These findings changed the central question from how to replace dopamine to why distinct susceptible neural systems accumulate pathological α-synuclein and progressively deteriorate.

The extraordinary expansion of the literature since 2000 reflects this conceptual broadening. PD is now investigated across epidemiology, prodromal markers, genetics, immunity, proteostasis, circuit dysfunction and regenerative medicine. Nevertheless, the field’s greatest achievement may not be any isolated technology. It is the replacement of a unitary, late-stage clinical construct by a multidimensional disease model in which etiology, molecular pathology, neuronal dysfunction and clinical expression are related but not interchangeable.

## Epidemiology: the Parkinson pandemic

PD is the second most common neurodegenerative disorder and its prevalence rises steeply with age. Contemporary estimates show pronounced variation by age, sex, geography and ancestry; men are affected more often than women, while under-ascertainment remains substantial in many low- and middle-income settings (Ben-Shlomo et al. 2024). The Global Burden of Disease analysis documented a more than twofold increase in the number of people living with PD between 1990 and 2016 (GBD 2016). Demographic ageing explains much, but probably not all, of this strong increase. Declining competing mortality, improved recognition and substantial environmental as well as climate change may contribute. Projections indicate a continuing sharp rise toward 2050, making prevention and scalable care urgent priorities (Su et al. 2025).

The phrase “Parkinson pandemic” is therefore not rhetorical: it describes a rapidly expanding neurological burden (Dorsey and Bloem 2018). Yet prevalence must not be confused with incidence, and some trends remain uncertain in regions with incomplete epidemiological data. Future studies must combine representative population studies with exposure, genomic and biomarker data, while addressing diagnostic inequity.

## From motor syndrome to multisystem disease

Bradykinesia together with rigidity or resting tremor remains the clinical core of parkinsonism and anchors the present Movement Disorder Society diagnostic criteria (Postuma et al. 2015). During the past 25 years, however, non-motor symptoms (NMS) have moved from the periphery of clinical description to the center of disease burden (Fig. 1). Hyposmia, constipation, depression, pain, autonomic dysfunction, cognitive impairment and sleep disorders such as REM (rapid eye movement) sleep behavior disorder (RBD) may precede motor diagnosis, accompany all disease stages and frequently have a very high impact on quality of life (Schapira et al. 2017).

This shift has two consequences. Clinically, assessment and treatment must be holistic rather than solely restricted to motor symptoms. Biologically, PD is currently understood as a multisystem disorder involving central and peripheral autonomic circuits, the enteric nervous system, olfactory pathways and widespread cortical-subcortical networks. Non-motor symptoms are not merely bystander of an advanced nigrostriatal loss; the majority of NMS is an early mirror of different underlying disease processes.

## Prodromal disease and biological staging

The recognition of a long prodromal interval created a probabilistic framework in which markers such as polysomnography-confirmed RBD, hyposmia, subtle motor signs and abnormal dopaminergic imaging modify an individual’s likelihood of prodromal PD. This was formalized in the MDS research criteria for prodromal PD and subsequent updates (Heinzel et al. 2019). These criteria remain research instruments: they estimate risk and should not be used to label asymptomatic individuals without appropriate counselling and validated interventions.

In 2024, two frameworks proposed a more radical transition from clinical diagnosis to biological classification. The neuronal α-synuclein disease integrated staging system (NSD-ISS) combines pathological neuronal α-synuclein, dopaminergic neuronal dysfunction and clinical impairment (Simuni et al. 2024). The SynNeurGe criteria classify individuals along three axes: pathological α-synuclein (S), neurodegeneration (N) and pathogenic genetics (G), followed by clinical characterization (Höglinger et al. 2024). Their value lies in separating biological state from phenotype and enabling mechanism-enriched trials. Their limitations are equally important: assay accessibility, incomplete sensitivity in genetic subgroups, uncertain longitudinal transitions and the ethical consequences of identifying disease prior to the onset of motor symptoms. For now, these classification systems are important frameworks, but rather of complementary nature and do not replace expert clinical diagnosis.

## Imaging parkinsonian syndromes

Conventional structural MRI remains primarily a tool to identify alternative or additional pathology. Over the past two decades, susceptibility-sensitive imaging, neuromelanin-sensitive MRI, diffusion metrics and quantitative morphometry have improved visualization of the nigrosome, the substantia nigra and distributed disease patterns. Machine-learning classifiers can combine regional measures to distinguish PD from multiple system atrophy (MSA) and progressive supranuclear palsy, but high performance in curated cohorts does not guarantee validity across scanners, disease stages and clinical populations.

Dopamine-transporter single-photon computed tomography (SPECT) and presynaptic dopaminergic positron emission tomography (PET) establish nigrostriatal dysfunction and can separate degenerative parkinsonism from many non-degenerative mimics, but they do not reliably distinguish PD from atypical degenerative parkinsonian syndromes. A selective α-synuclein PET would be a decisive break through, yet remains technically difficult because of low target density, intracellular localization and the need for conformer selectivity. [18F] ACI-12589 demonstrated binding compatible with α-synuclein pathology in MSA, but not a diagnostic separation of PD from controls (Smith et al. 2023). Thus, imaging is increasingly quantitative and pathophysiological, but no current scan modality constitutes a universal molecular ground truth read-out of PD.

## Progression, trajectories and phenotypes

PD progression is nonlinear and heterogeneous. Group averages obscure clinically meaningful trajectories: some patients retain relatively preserved gait and cognition for many years, whereas others develop early falls, autonomic failure or dementia. Age at onset, baseline phenotype, cognitive status, RBD, genetic architecture and comorbidity shape progression, but current models remain far from perfect at an individual level. Data-driven clustering has identified slow, intermediate and fast-progressing subgroups, although clusters depend strongly on selected variables, follow-up duration and analytic assumptions.

The brain-first/body-first hypothesis offers a mechanistically attractive account partially reflecting phenotypic heterogeneity. In a body-first pattern, pathology may arise in peripheral autonomic structures and ascend through connected neural pathways, often associated with RBD and early autonomic dysfunction; in a brain-first pattern, initial pathology may be more asymmetric and centrally restricted. Both models should be viewed as a testable spectrum rather than a rigid dichotomy. Phenotyping becomes most useful when it predicts biology, prognosis or treatment response; descriptive labels alone risk reclassifying variability without explaining it.

## Gene-environment interaction: pesticides

PD is predominantly multifactorial. Occupational and residential pesticide exposures provide one of the strongest and most actionable environmental signals, although exposure assessment, latency and correlated agricultural factors complicate causal inference. In the Agricultural Health Study, paraquat and rotenone exposure were associated with PD; both compounds map onto experimentally relevant mechanisms, oxidative stress and mitochondrial complex I dysfunction, respectively (Tanner et al. 2011). Associations may be modified by susceptibility genes, protective equipment and work practices, underscoring that “pesticides” are not a single exposure class.

The recognition of pesticide-associated PD as an occupational disease in some western societies marks an important translation of epidemiological evidence. Scientifically, the priority is to move from broad categories to molecule-specific, dose-resolved exposure histories and gene-environment interaction models. Clinically, careful occupational history is warranted, while prevention must emphasize exposure reduction rather than waiting for complete mechanistic certainty.

## Dopaminergic therapy: sustained success and remaining limits

Levodopa remains the most effective treatment for bradykinesia and rigidity (Fig. 1). Advances since 2000 have refined rather than displaced dopaminergic replacement: individualized combinations with decarboxylase inhibitors, dopamine agonists, monoamine oxidase-B inhibitors and catechol-O-methyltransferase inhibitors extend benefit and reduce a broad spectrum of motor fluctuations i.e. such as *off* time or dykinesias. The major conceptual advance has been the recognition of pulsatile delivery against a progressively shrinking buffering capacity of presynaptic vesicles. This has driven the development of sustained long-acting oral formulations, continuous intestinal or subcutaneous levodopa and apomorphine delivery.

Treatment must balance motor control with dyskinesia, hallucinations, orthostatic hypotension, somnolence and impulse-control disorders. Device-aided approaches such as infusion therapies and deep brain stimulations are highly effective in selected patients but do not slow down or halt neurodegeneration. Twenty-five years of therapeutic development have therefore strengthened a dual message: symptomatic therapy can be transformative, yet dopamine replacement is not able to address the full spectrum of a multisystem disorder such as PD or prevent its progression (Fig. 1).

## α-Synuclein: pathogenesis and diagnostics

α-Synuclein has become the predominant molecule connecting PD genetics, pathology and biomarker development (Fig. 1). SNCA point mutations and locus multiplication demonstrate that altered sequence or increased dosage is sufficient to cause disease; triplication established a direct gene-dose relationship (Singleton et al. 2003). At presynaptic terminals, physiological α-synuclein participates in synaptic-vesicle homeostasis. Pathological species undergo conformational conversion, β-sheet enrichment, post-translational modifications and assembly into oligomers and fibrils. Serine−129 phosphorylation is strongly enriched in Lewy bodies, although whether it is causal, compensatory or a marker of aggregation remains disputed.

The Braak staging scheme proposed a stereotyped, caudo-rostral distribution of Lewy pathology (Braak et al. 2003). It was a conceptual breakthrough and stimulated the propagation hypothesis, but obviously does not capture every patient (Braak H, Braak E 2000). Experimental systems support cell-to-cell transfer and templated seeding through several routes, while clinicopathological heterogeneity suggests multiple origins and patterns. Distinct α-synuclein conformers may contribute to phenotypic differences among PD, dementia with Lewy bodies and MSA.

Seed amplification assays (SAAs) are the most consequential diagnostic advance in the field of PD research. By amplifying seeding-competent α-synuclein from biospecimens, cerebrospinal-fluid SAAs achieve high diagnostic performance in many cohorts. In the Parkinson’s Progression Markers Initiative, SAA positivity captured biological heterogeneity and was detectable in many prodromal participants (Siderow et al. 2023). Skin-based phosphorylated α-synuclein and peripheral-tissue assays provide complementary routes, while blood, saliva, stool and urine remain to be systematically explored in the future. SAAs identify a seeding activity, not its anatomical burden, and binary results do not yet reflect the rate of progression. Standardization, reference materials and longitudinal validation are prerequisites for routine use and treatment monitoring.

## Disease modification: immunotherapy and lysosomal proteostasis

The significance of extracellular transfer made an anti-α-synuclein immunotherapy a rational strategy. However, the SPARK trial of cinpanemab and PASADENA trial of prasinezumab did not meet their primary clinical endpoints in early PD (Lang et al. 2022; Pagano et al. 2022). Post-hoc and open-label analyses of prasinezumab have generated signals in rapidly progressing subgroups and form the hypothesis-generating basis for the current PARAISO trial (Pagano et al. 2024). This follow up trial opens a new avenue by trying to validate α-synuclein as a target. In general, α-synuclein immunotherapies expose unresolved issues of epitope, intracellular versus extracellular target access, brain penetration, pharmacodynamic measurement, disease stage and biological enrichment. Future studies must incorporate α-synuclein as biomarker and pre-specified progression strata.

Inflammatory biology offers additional targets in PD. α-Synuclein-reactive T-cell responses and microglial-astrocytic interactions demonstrate adaptive and innate immune engagement, but it remains unclear when inflammation initiates injury, amplifies proteinopathy or supports repair. Broad immunosuppression is unlikely to provide the precision required; cell state, timing and compartment will matter.

Genetics has independently converged on the autophagy-lysosomal pathway (Fig. 1). Heterozygous GBA1 variants are among the strongest common genetic risk factors for PD and are associated, on average, with earlier onset and faster cognitive and motor progression (Sidransky et al. 2009). Reduced enzyme activity of GBA1 and α-synuclein accumulation are able to reinforce one another through a pathogenic feedback loop. Therapeutic approaches include chaperones, substrate reduction, enzyme activators and gene therapy. Yet, lysosomal restoration will require evidence of central target engagement and may be most effective in biomarker-defined subgroups. The larger lesson is that proteostasis is not an end-stage housekeeping failure; it is rather a genetically anchored therapeutic axis to maintain the dynamic regulation of a balanced, functional proteom.

## Cell therapy, stem-cell models and organoids

Cell replacement illustrates both the ambition and the caution of contemporary PD research (Fig. 1). Fetal ventral-mesencephalic grafts established that transplanted dopaminergic neurons survive, reinnervate striatum and provide prolonged benefit, but randomized trials yielded variable efficacy and graft-induced dyskinesia (Olanow et al. 2003). Lewy body pathology detected years later in grafted neurons provided astonishing evidence of host-to-graft propagation (Li et al. 2008; Kordower et al. 2008).

Pluripotent stem cells now permit standardized production of authentic human ventral midbrain dopaminergic progenitors (Fig. 1). A personalized autologous induced pluripotent stem cells (iPSC) derived graft showed survival and increased fluorodopa uptake in a proof-of-concept case (Schweitzer et al. 2020). Embryonic-stem-cell and iPSC-derived products have subsequently entered early clinical testing. The essential questions are long-term safety, purity, dose, graft integration, dyskinesia, immune management and vulnerability to the host disease. Cell replacement restores a distinct neuronal population; it is not, by itself, a causal treatment of a multisystem synucleinopathy.

The impact of iPSCs related technology may be even broader. Patient-derived neurons and isogenic gene-corrected controls connect genotype to cellular phenotypes and enable an individualized compound testing. Midbrain and 3-D organoids add multicellular architecture, maturation and circuit context, while enteric and vagal models have the potential to interrogate body-brain interactions. Their limitations such as developmental immaturity, batch variability, incomplete vasculature and immune representation must be addressed in the near future. The most promising use of these human multicellular organoids will integrate reductionist isogenic models with longitudinal human biomarker cohorts rather than treating organoids as miniature replicas of late-life PD.

## Outlook: the next 25 years

In the following section, important future developments are lined out in the context of present knowledge, however are of speculative nature to a certain extent and based upon personal judgements. By 2050, PD treatment may begin prior to motor symptoms or motor-based diagnosis supported by basic science evidence that molecular and biological detection can be linked to prognosis (Fig. 1). α-Synuclein seed amplification assays of the CSF or of other body tissues and fluids identify a crucial, biological feature of PD, but do not reliably establish its anatomical extent, rate of progression or treatment response. Future staging will therefore need to combine modern α-synuclein hybrid PET/ MRI imaging technologies, individual neuropathology, circuit functions and longitudinal outcomes, validated across genetic backgrounds and different populations (Siderow et al. 2023; Simuni et al. 2024). Indeed, baseline assay kinetics were not able to consistently predict five-year motor or cognitive outcomes (Belete et al. 2026).

The decisive advance in the clinical field of PD would be an oral treatment that proved to slow down disease progression, potentially administered in particular during prodromal or early symptomatic disease stages. Trials may preferentially select participants by genetic risk, α-synuclein biology and the neural pathway targeted. Thus, therapies addressing protein aggregation, lysosomal and mitochondrial dysfunction or neuroimmune responses must demonstrate central target engagement and long-term clinical benefit. Therapeutic prevention trials in high-risk groups will require reliable prediction of individual prognoses and a favorable balance of clinical benefit and adverse treatment-emergent adverse events.

Symptomatic treatment may become more responsive to daily fluctuations through non-body worn sensor-based assessments i.e. of motor functions, continuous drug delivery with sustained benefits or adaptive deep brain stimulation. In particular, a blinded feasibility trial supports future studies of adaptive deep brain stimulation, although long-term patient-relevant benefit remains uncertain at present (Oehrn et al. 2024). Stem-cell-derived grafts may restore striatal dopamine in highly selected patients if controlled studies confirm durable safety and efficacy. At present, early iPSC grafts results do not establish disease modification or address non-motor symptoms (Sawamoto et al. 2025).

Patient-derived cellular models such as 3-D organoids may connect the individual genotype and exposure to treatment response if their findings consequently replicate in clinical cohorts. Reducing environmental risks and a broad access to public healthcare will matter just as much. In summary, the goal for the next quarter century is to delay onset where possible, to slow down progression in biologically defined groups and preserve function throughout the multisystem course of PD.

## Conclusion

Between 2000 and 2025, PD research progressed from a largely clinicopathological and dopamine-centred framework to an integrated view of a heterogeneous, systemic and biologically measurable disorder. The field now detects pathological α-synuclein species in patients, define genetic and lysosomal subgroups, model patient-specific disease in human cells and manufacture dopaminergic progenitors for transplantation. Yet disease modification remains an overall crucial goal. The decisive next step is alignment: biological definition with clinical phenotype, intervention mechanism with demonstrable target engagement, and trial duration mirroring the timescale of PD-associated neurodegeneration. The central achievement of the past 25 years is therefore not the discovery of a single final pathway, but the creation of a framework capable of matching the right patient to a distinct target at its individual disease stage for the next decades to come.

## Data Availability

No datasets were generated or analysed during the current study.
